# Rhein inhibits *Chlamydia trachomatis* infection by regulating pathogen-host cell

**DOI:** 10.3389/fpubh.2022.1002029

**Published:** 2022-09-26

**Authors:** Xueying Yu, Qingqing Xu, Wentao Chen, Zhida Mai, Lijun Mo, Xin Su, Jiangli Ou, Yinyuan Lan, Heping Zheng, Yaohua Xue

**Affiliations:** ^1^Department of Clinical Laboratory, Dermatology Hospital, Southern Medical University, Guangzhou, China; ^2^Department of Clinical Laboratory, Shanghai Fourth People's Hospital Affiliated to Tongji University School of Medicine, Shanghai, China; ^3^Guangzhou Key Laboratory for Sexually Transmitted Diseases Control, Guangzhou, China

**Keywords:** *Chlamydia trachomatis*, antibacterial activity, rhein, host-directed therapy, azithromycin, *in vivo*

## Abstract

The global incidence of genital *Chlamydia trachomatis* infection increased rapidly as the primary available treatment of *C. trachomatis* infection being the use of antibiotics. However, the development of antibiotics resistant stain and other treatment failures are often observed in patients. Consequently, novel therapeutics are urgently required. Rhein is a monomer derivative of anthraquinone compounds with an anti-infection activity. This study investigated the effects of rhein on treating *C. trachomatis* infection. Rhein showed significant inhibitory effects on the growth of *C. trachomatis* in multiple serovars of *C. trachomatis*, including D, E, F and L1, and in various host cells, including HeLa, McCoy and Vero. Rhein could not directly inactivate *C. trachomatis* but could inhibit the growth of *C. trachomatis* by regulating pathogen-host cell interactions. Combined with azithromycin, the inhibitory effect of rehin was synergistic both *in vitro* and *in vivo*. Together these findings suggest that rhein could be developed for the treatment of *C. trachomatis* infections.

## Introduction

*Chlamydia trachomatis (C. trachomatis)*, a Gram-negative bacterial pathogen, is a causative agent of sexually transmitted infections in humans. There are an estimated 127 million new cases of *C. trachomatis* genital infection annually worldwide (1). *C. trachomatis* is classified into 15 serovars, with serovars D to K causing urinary or genital tract infections and serovars L1 to L3 being associated with lymphogranuloma venereum (2, 3). The developmental cycle of *C. trachomatis* is unique and biphasic, featuring an infective, metabolically inactive, elementary body (EB) and a metabolically active, intracellular, reproductive reticular body (RB) (4). Many individuals infected with *C. trachomatis* are asymptomatic, but chlamydia infections can have serious consequences. Untreated or recurrent chlamydial urogenital infections can lead to severe complications such as pelvic inflammatory disease and infertility (5). Genital infection with *C. trachomatis* also facilitates other sexually transmitted infections such as HIV (6) and human papillomavirus (7, 8). Thus, the potential risk chlamydia poses to human health should not be underestimated.

Currently, 1 g azithromycin (AZM) or 100 mg doxycycline twice a day for 7 days is the unanimously recommended, first-line treatment for uncomplicated *C. trachomatis* infections of the urogenital tract in the United States (9), China (10, 11), Europe (12), and Australia (13). Nevertheless, antibiotic resistance or treatment failure is not uncommon in *C. trachomatis* infections (10, 14, 15). For example, AZM treatment failure has been reported in 5% to 23% of *Chlamydia*-positive men with non-gonococcal urethritis and women with cervicitis not at risk of reinfection (16). A partner-treatment study reported that 8% of women had persistent chlamydial infection despite stating they had no sexual contact after treatment (17). These treatment failures may be due to resistance in members of the genus *C. trachomatis* and persistent infection. The *tet (M)* gene confers resistance to tetracycline antibiotics, while mutations in the 23S rRNA gene confer resistance to macrolides (18–20). The rates of 23S rRNA gene mutations and the abundance of *tet (M)* in *C. trachomatis* were higher in a treatment-failure group than in a treatment-success group (21). Furthermore, following exposure to antimicrobial drugs at sub-inhibitory concentrations, *C. trachomatis* can transform into a surviving reticulate with an almost persistently quiescent metabolism, which further increases the resistance to antibiotics (22). The emergence of antibiotic resistance and treatment failure indicated the need to identify novel anti-chlamydial agents.

**Figure 1:**
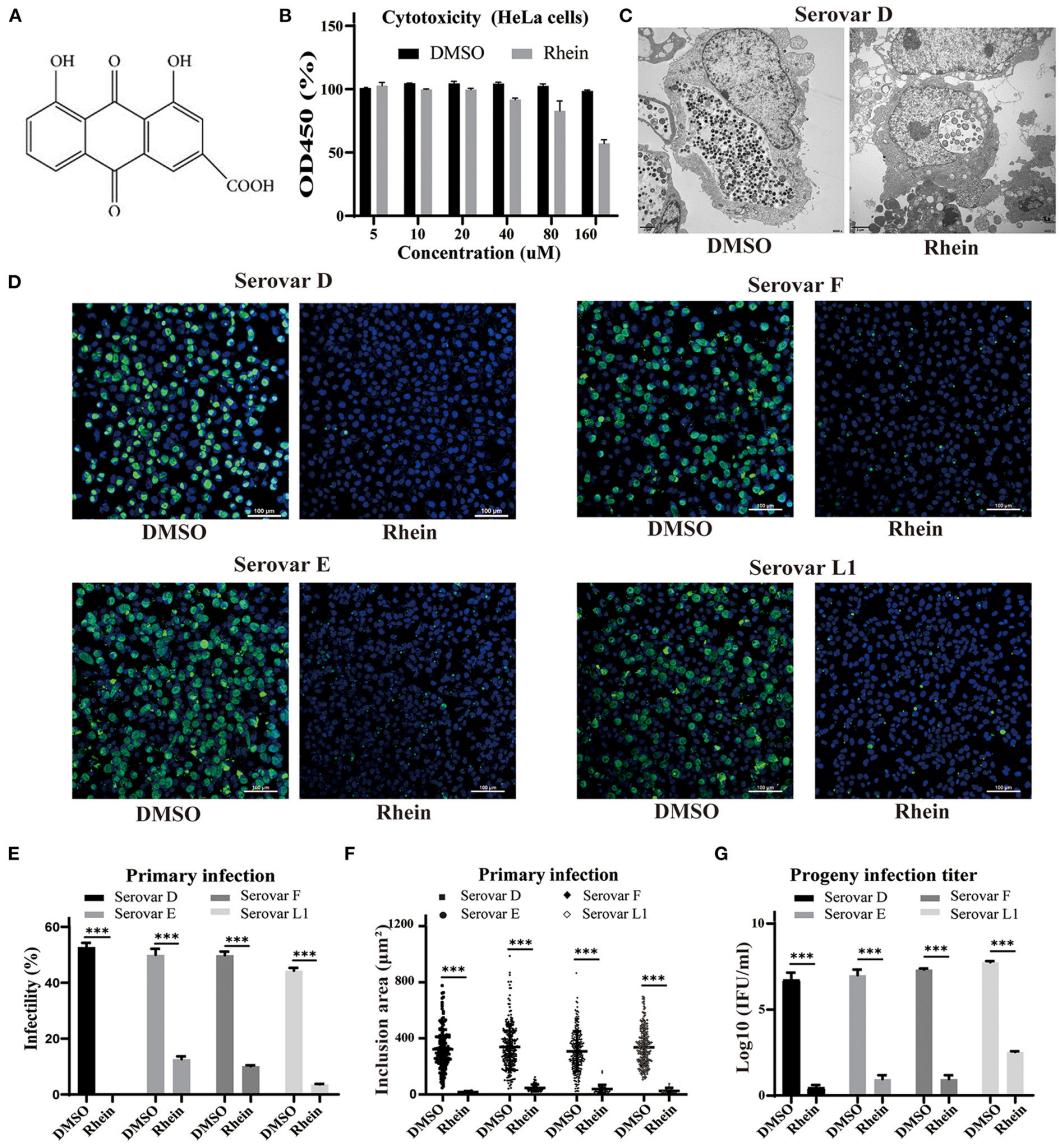
Rhein efficiently inhibited *C. trachomatis* replication. **(A)** Chemical structure of rhein. **(B)** Cytotoxic effect of rhein on HeLa cells assessed using a Cell Counting Kit-8. **(C)** Representative transmission electron micrographs of HeLa cells infected with *C. trachomatis* serovar D and exposed to 40 μm rhein or DMSO. Scale bars, 2 μm. **(D)** Immunofluorescent staining of HeLa cells infected with *C. trachomatis* serovars D, E, F, and L1 and exposed to 40 μM rhein for 48 h. *C. trachomatis* inclusions were stained with FITC-conjugated MOMP antibody (green) and nuclei were counterstained with DAPI (blue). Fluorescent images were captured on a confocal microscope at ×200 magnification. Scale bars, 100 μm. **(E)** Infectivity. **(F)** Inclusion area. **(G)** Infectious progeny titer. Data bars in b, e, and g represent the mean ± standard deviation. *p< 0.05, **p < 0.01, ***p< 0.001.

Phytochemicals have attracted attention in recent years because of their therapeutic potential against a wide variety of pathogenic microorganisms (23). Compounds extracted from biomaterials and phytochemicals include flavones (24), terpenoids (25), alkaloids (26), and essential oils (27), and many of these compounds have antimicrobial properties. In a previous study, rhubarb inhibited *C. trachomatis* infection (28). Rhein (4, 5-dihydroxyanthraquinone-2-carboxylic acid; Figure 1A) is a monomer primarily extracted from rhubarb (29, 30). This lipophilic, naturally occurring compound has numerous pharmacological properties, including antitumor, antioxidant, anti-inflammatory, antimicrobial, hepatoprotective, and nephroprotective activities (31). As an antimicrobial, rhein has effects against *Staphylococcus aureus* (32, 33), *Pseudomonas aeruginosa* (34), *Porphyromonas gingivalis* (35), and influenza virus (36), among others. In the current study, the effects of rhein on *C. trachomatis* replication in cell culture and in mice were investigated with the aim of determining whether rhein had potential as a novel therapeutic against *C. trachomatis* infections.

## Materials and methods

### Cell culture and *C. trachomatis* strains

Human epithelial carcinoma cells (HeLa) (ATCC CCL-2.1), mouse fibroblast cells (McCoy) (ATCC CTL-1696) provided by Dr. Lifang Jiang (Sun Yat-sen University, China) and African green monkey kidney cells (Vero) (CCTCC GDC062) were maintained in Dulbecco's modified Eagle's medium (Gibco, St. Louis, MO, USA) with 10% heat-inactivated fetal bovine serum (Gibco) at 37°C in an incubator supplied with 5% CO_2_ (Sanyo, Tokyo, Japan). *C. trachomatis* serovars D, E, F and L1 were provided by Dr. Joke Spaargaren (Public Health Laboratory of the Municipal Health Service of Amsterdam, Netherlands).

To obtain sufficient quantity of *C. trachomatis*, confluent HeLa cells were infected with *C. trachomatis* and centrifuged for 60 min at 1,000 × *g*. After centrifugation, the supernatants were replaced with 1 ml maintenance medium supplemented with 1.0 μg/ml cycloheximide (MedChemExpress, Monmouth Junction, NJ, USA). At 48 hpi, infected cells were sonicated and resuspended in sucrose–phosphate–glutamate. Stocks were divided into small aliquots and stored frozen at −70°C.

### Compounds and drugs

Rhein (MedChemExpress) and AZM (North China Pharmaceutical Group Corporation, Hebei, China) were dissolved according to the manufacturers' instructions and stored at −70°C. DMSO (Sigma, St. Louis, USA) was stored at 4°C.

### Cytotoxicity assays with rhein

Cytotoxicity of rhein in HeLa cells was assessed using a Cell Counting Kit-8 (CCK-8) (Dojindo, Tokyo, Japan) according to the manufacturer's instructions. Briefly, HeLa cells were seeded at 1 × 10^4^ cells per well in 96-well plates and incubated overnight. Cell monolayers were exposed to various concentrations of rhein (0, 5, 10, 20, 40, 80, and 160 μM) for 48 h, then the CCK-8 kit was utilized, measuring the absorbance of the cells, and the results were expressed as percent viable cells.

### Immunofluorescent staining

Confluent HeLa cells were centrifuged at 1,000 × *g* with *C. trachomatis* for 1 h and then placed at 37°C in an incubator supplied with 5% CO_2_ for 1 h. The medium was then changed to maintenance medium in the presence of 40 μM rhein or DMSO. Infected HeLa cells were cultured on cell slides for 48 h and fixed with 4% paraformaldehyde for 20 min at room temperature. Cells were permeabilized with 0.1% Triton X-100 for 20 min at room temperature and were then incubated with 1% bovine serum albumin in phosphate-buffered saline with Tween (PBS + 0.1% Tween 20) for 60 min to block non-specific binding of the antibodies. Cells were incubated with a fluorescein isothiocyanate (FITC)-conjugated antibody against the major outer membrane protein (MOMP) of *C. trachomatis* (Abcam, Cambridge, UK) and were then counterstained with DAPI (Solarbio, Beijing, China).

### Confocal microscopy

Samples were examined under a confocal microscopy at ×200 magnification (Nikon, Tokyo, Japan) and the number of inclusions and nuclei were counted in 15 random fields from triplicate samples in each experiment. The number of inclusions and nuclei were used to assess infectivity by calculating the inclusion/nuclei percent with Nikon AR NIS 5.02.00 software. The software was also used to measure the area of inclusion bodies.

### Electron microscopy

Infected HeLa cells were centrifuged at 1,000 × *g* for 1 h and then placed at 37°C in an incubator supplied with 5% CO_2_ for 1 h. The medium was then changed to maintenance medium in the presence of 40 μM rhein or DMSO. At 48 hpi, cells treated with rhein or DMSO were collected, pelleted by centrifugation at 1,000 × *g* for 5 min, and were then embedded and examined by transmission electron microscopy (Japan Electron Optics Laboratory, Tokyo, Japan).

### Titer of infectious progeny assay

*C. trachomatis*-infected cells were collected and sonicated. EBs were released from the cells and used to infect fresh cell monolayers. Total inclusions were counted, and numbers of inclusion-forming units (IFU/ml) were calculated at 48 hpi.

### Direct interaction assay

EBs of *C. trachomatis* were co-incubated with 40 μM rhein for 12, 24, 36, or 48 h at 4°C before infection (37); DMSO was used as a positive control. *C. trachomatis* was washed with PBS to remove the residual rhein and was then used to infect HeLa cells in 24-well plates. At 48 hpi, cells were fixed with paraformaldehyde and observed by confocal microscopy.

### Influence-on-cell assay

HeLa cells were seeded in 24-well plates at 1 × 10^5^ cells/well and 40 μM rhein was added to the culture medium and incubated for 24 h. Cell monolayers were washed three times with PBS, then the pretreated cells were infected with *C. trachomatis*; DMSO treatment served as a positive control. At 48 hpi, cells were stained with MOMP antibody and observed using confocal microscopy.

### Influence-on-adsorption assay

HeLa cells were infected with *C. trachomatis* and simultaneously exposed to 40 μM rhein in the culture medium; a control group received the equivalent amount of DMSO. The culture plate was centrifuged at 1,500 × *g* for 1 h and then placed at 37°C in an incubator supplied with 5% CO_2_ for 1 h. The medium containing rhein was then discarded, and cells were washed with PBS three times before addition of the maintenance medium. Immunofluorescence staining was conducted at 48 hpi.

### Western blotting

Treated cells were incubated for 0, 12, 24, 36, or 48 h, then the cellular proteins were lysed by RIPA (Invitrogen, 89900) supplemented with a protease and phosphatase inhibitor cocktail (Invitrogen, 78440), and incubated with SDS-PAGE loading buffer (Reducing) (Cwbio, CW0027) at 100°C for 10 min. Antibodies used for western blotting were as follows: anti-RSK1 p90 (phospho T359 + S363) antibody (1:1,000, ab32413, Abcam), anti-RSK1 p90 antibody (1:1,000, ab32114, Abcam), anti-Phospho-p44/42 MAPK (Erk1/2) (1:1,000, 4370S, Cell signaling), anti- p44/42 MAPK (Erk1/2) (1:1,000, 4695S, Cell signaling), anti-cHSP60 (1:2,000, sc-57840, Santa Cruz), anti-GAPDH (1:1,0000, ab181602, Abcam), anti-rabbit IgG-HRP-linked antibody (1:5,000, 7074S, Cell signaling), and anti-mouse IgG-HRP-linked antibody (1:5,000, 7076S, Cell signaling). Blots were imaged on a ChemiDoc MP Imaging System (Bio-Rad).

### Animals

Female BALB/c mice (4–6-week-old) were purchased from the Southern Medical University (Guangzhou, China). At 10 and 7 days before infection, all mice were injected subcutaneously with 2.5 mg medroxyprogesterone acetate (Bayunshan Pharmaceutical Company, Guangzhou, China) to synchronize estrus (38). After treatment, the mice were vaginally infected with 1 × 10^7^ *C. trachomatis* IFU or an equal volume of sucrose–phosphate–glutamate. Experiments were conducted in the Experimental Animal Center of South China Agricultural University and in accordance with the National Institutes of Health Guide for the Care and Use of Laboratory Animals. All procedures performed in studies involving experiments on animals were approved by the Ethics Committee of South China Agricultural University (SCAU, Guangzhou, China and approval number: 2020c035).

### Drug treatment *in vivo*

Mice were divided into five groups: negative control, positive control, rhein treatment, AZM treatment and rhein + AZM combined treatment. Rhein was dissolved in DMSO at 10 mg/ml, and AZM was dissolved in ethanol at 0.084 mg/ml. Mice were treated with 120 mg/kg rhein, 1.0 mg/kg AZM or a combination of 120 mg/kg rhein and 1.0 mg/kg AZM in 0.5% carboxymethylcellulose sodium (CMC-Na) once daily by gavage from day 4 to day 10. Control mice were gavaged with 0.5% CMC-Na. Vaginal swabs were taken for cell culture on day 4 (before gavage), day 7 and day 10 after infection, and the number of inclusions were measured.

### Statistical analyses

GraphPad Prism 8 (GraphPad Software, La Jolla, CA, USA) was used to generate graphs, and statistical analyses were conducted using SPSS 15.0 (SPSS Inc., Chicago, IL, USA). Quantitative data are presented as mean ± standard deviation. The Shapiro–Wilk test was used to test the normality of quantitative data. Fisher's exact test and Bonferroni's multiple comparisons were used to assess infectivity. Kruskal–Wallis and Dunn's multiple comparisons tests were used to evaluate the area of inclusions. An unpaired *t*-test was used to analyze the difference in EB titer between groups. *P*-values were calculated using one-way ANOVA followed by Bonferroni correction for multiple comparisons. A nonparametric Wilcoxon test was used for mouse model statistics. Differences were considered significant at *P* <0.05 (^*^), *P* <0.01 (^**^) and *P* <0.001 (^***^).

## Results

### Rhein effectively inhibited *C. trachomatis* replication

Cell viability was approximately 95% in samples exposed to 40 μM rhein (Figure 1B). The anti-chlamydial effects of rhein were investigated in HeLa cells infected with the more prevalent serovars of *C. trachomatis* (serovars D, E and F) and the L1 serovar that can lead to venereal lymphogranuloma (39). A few aberrant RBs were observed by transmission electron microscopy in HeLa cells infected with *C. trachomatis* serovar D and treated with 40 μM rhein, compared with many small, mature EB particles within the inclusion of DMSO-treated cells at 48 hpi (Figure 1C). Immunofluorescent staining revealed that the inclusion bodies became smaller and the infectivity, inclusion size and infectious progeny decreased in the presence of 40 μM rhein (Figures 1D–G). These results demonstrated that rhein effectively inhibited the growth and reproduction of different serovars of *C. trachomatis* in HeLa cells.

### The effect of rhein on *C. trachomatis* was dose- and time-dependent

**Figure 2:**
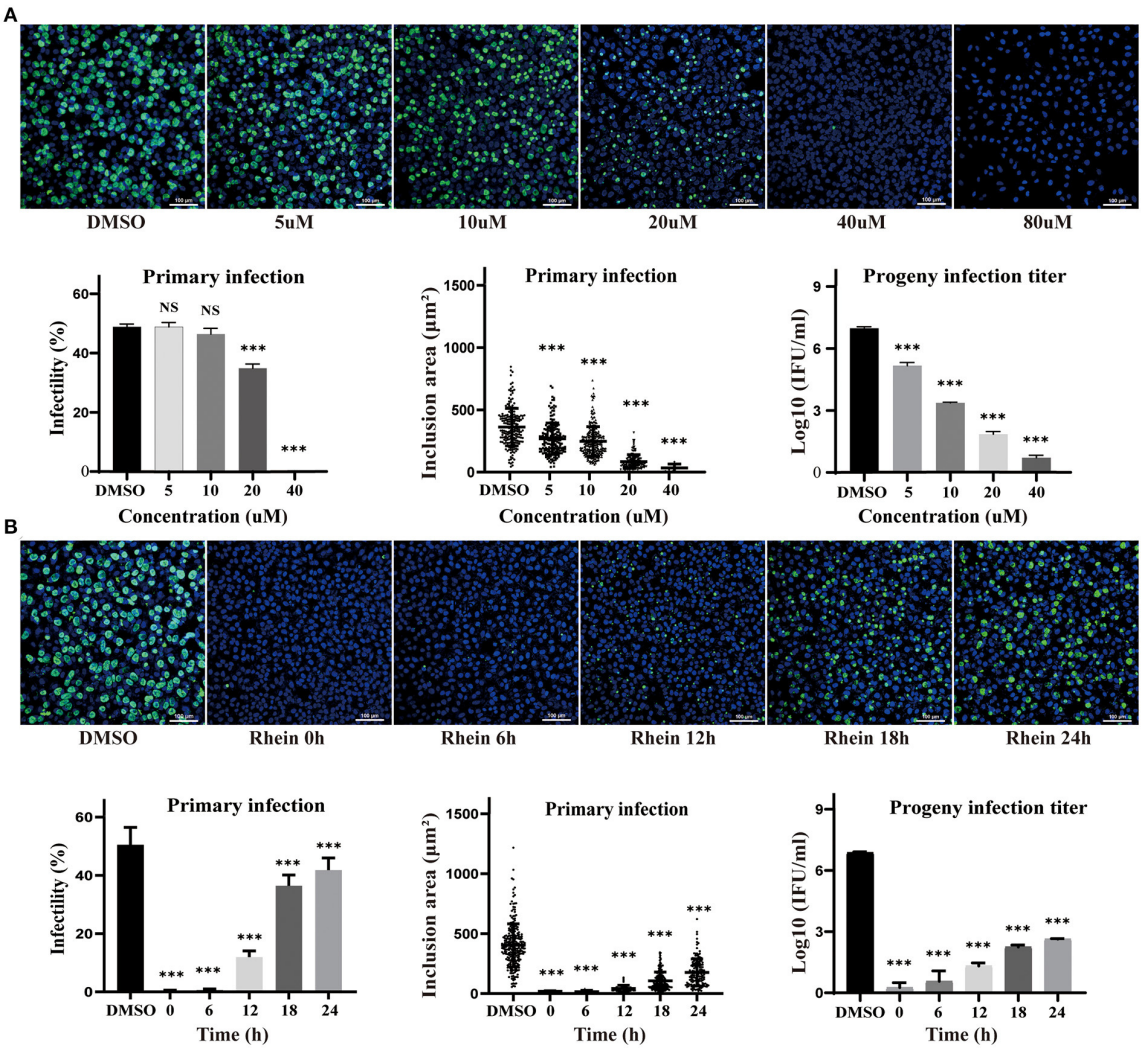
Inhibitory effects of rhein on *C. trachomatis* infection were dose- and time-dependent. **(A)** HeLa cells were infected with *C. trachomatis* serovar D at multiplicity of infection (MOI) 5 and were exposed to various concentrations of rhein (5, 10, 20, 40, and 80 μM) or DMSO for 48 h before fixation and immunostaining. **(B)** HeLa cells infected with *C. trachomatis* were exposed to rhein (40 μM) at 0, 6, 12, 18, and 24 hpi. Cells were fixed, and *C.trachomatis* were stained with a FITC-conjugated anti-MOMP antibody (green), while host cell nuclei were counterstained with DAPI (blue). Scale bars, 100 μm. Data bars in the graphs represent the mean ± standard deviation. NS, not significant; *p< 0.05, **p < 0.01, ***p< 0.001.

The effect of different concentrations of rhein (0, 5, 10, 20, 40, and 80 μM) on HeLa cells infected with *C. trachomatis* was examined, and the infectivity, inclusion area and infectious progeny are decreased in the presence of rhein in a dose-dependent manner (Figure 2A). HeLa cells infected with *C. trachomatis* were also exposed to 40 μM rhein at various time points (0, 6, 12, 18, and 24 h) after infection. *C. trachomatis* inclusions were larger and more numerous with rhein exposure at 24 hpi compared with rhein exposure at 0 hpi (Figure 2B). The titer of infectious progeny also increased with the delay in exposure to rhein (Figure 2B). These findings indicated that rhein inhibited the replication of *C. trachomatis* in a dose- and time-dependent manner, and suggested that the earlier cells are treated with rhein, the better the inhibition of *C. trachomatis*.

### Rhein did not directly inactivate *C. trachomatis* elementary bodies

**Figure 3:**
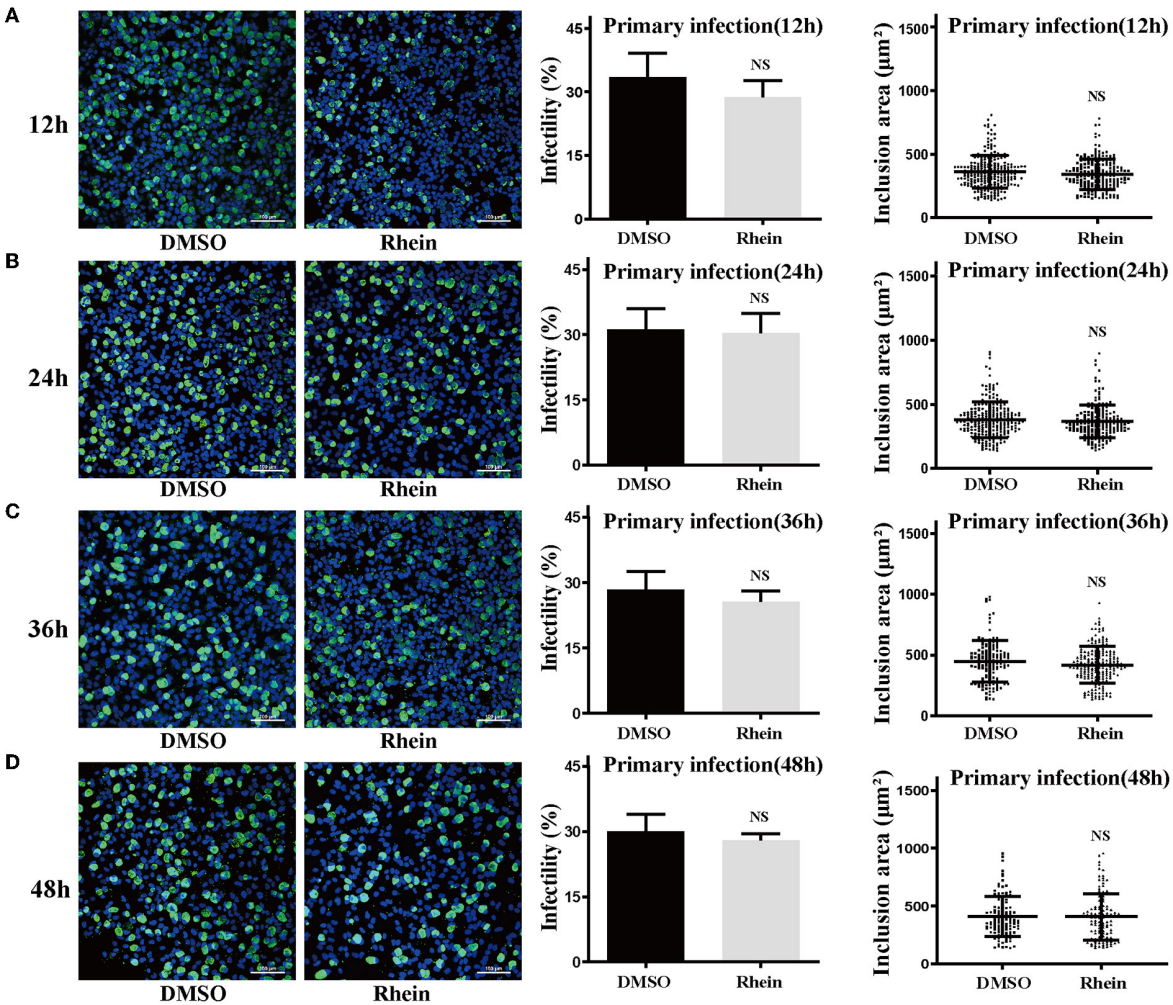
Pretreatment with rhein did not impair *C. trachomatis* particles. Elementary bodies of *C. trachomatis* were respectively co-incubated with 40 μM rhein for 12, 24, 36, or 48 h at 4°C before infection. At 48 hpi, *C. trachomatis* inclusions, infectivity, and inclusion areas were observed by confocal microscopy. **(A)** 12 h; **(B)** 24 h; **(C)** 36 h; and **(D)** 48 h. Images were captured at ×200 magnification. Scale bars, 100 μm. Data represent the mean ± standard deviation of triplicate samples. NS, not significant. *p< 0.05, **p < 0.01, ***p< 0.001.

Rhein and other anthraquinone drugs, including emodin and aloe-emodin, have been extracted from rhubarb. Emodin and aloe-emodin have antibacterial or virucidal activity by destroying the envelope of bacteria or viruses (40–43). Rhein was previously demonstrated to directly inhibit the growth of *S. aureus* (33). To determine whether rhein could directly impair *C. trachomatis* activity, 40 μM rhein was co-incubated with *C. trachomatis* serovar D for 12, 24, 36, and 48 h, respectively (36, 40). The infectivity and inclusion area of *C. trachomatis* exposed to rhein were not significantly different from those of the corresponding DMSO control (*P* > 0.05; Figure 3). This suggested that rhein did not directly inactivate *C. trachomatis* EBs.

### Rhein inhibited *C. trachomatis* through regulation of host cells

**Figure 4:**
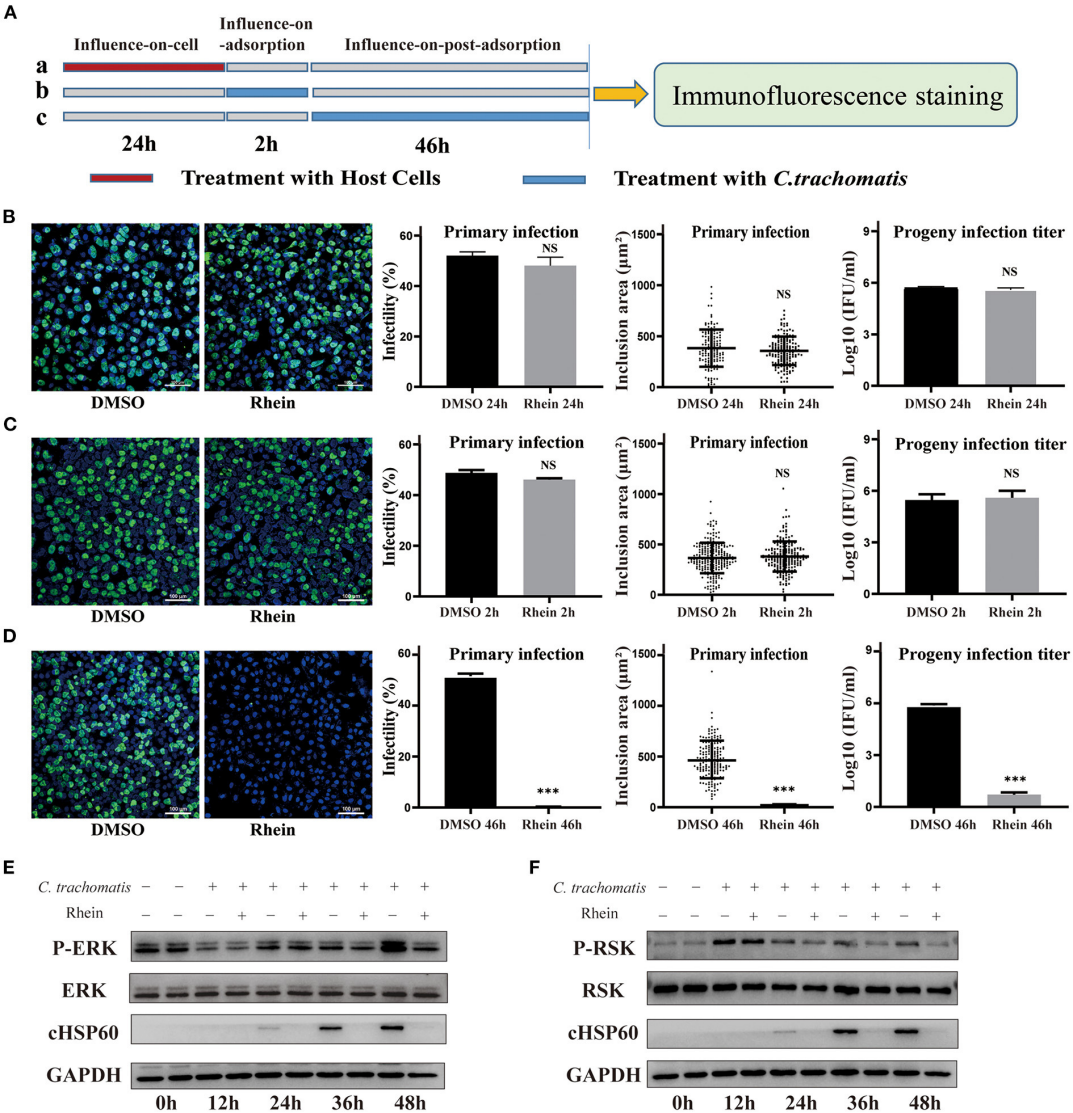
Rhein inhibited *C. trachomatis* infection by regulating host cells. **(A)** Three treatment conditions (each row is a treatment): **(A)** influence-on-cell, cells were pretreated with 40 μM rhein for 24 h; **(B)** influence-on-adsorption, cells were exposed to 40 μM rhein for 2 h during the period of adsorption; **(C)** influence-on-post-adsorption, cells were exposed to 40 μM rhein for 46 h after adsorption. **(B–D)** Immunofluorescent images (×200 magnification), infectivity, inclusion area, and infectious progeny titer. DMSO was used as positive control. **(B)** Cells were pretreated with 40 μM rhein for 24 h (treatment a). **(C)** Cells were exposed 40 μM rhein for 2 h during the period of adsorption (treatment b). **(D)** Cells were exposed 40 μM rhein for 46 h after adsorption (treatment c). **(E)** Western blots of p-ERK, ERK, cHSP60 and GAPDH protein expression in *C. trachomatis*-infected cells with or without rhein at different time points post-infection. The bands were cropped from different parts of the same gel. **(F)** Western blots of p-RSK, RSK, cHSP60 and GAPDH protein expression in *C. trachomatis*-infected cells with or without rhein at different time points post-infection. The bands were cropped from different parts of the same gel. Data in the graphs represent the mean ± standard deviation of triplicate samples. NS, not significant; *p< 0.05, **p < 0.01, ***p< 0.001.

*C. trachomatis* is an obligate intracellular parasitic pathogen that needs to combine with host surface receptors to enter a cell. The pathogen then uses host cell nutrients to replicate and reproduce by regulating the interaction with the host cell (44). Rhein did not have a direct inactivation effect on *C. trachomatis*. To elucidate the potential inhibitory mechanism of rhein, a set of influence-on-cell, influence-on-adsorption, and influence-on-post-adsorption assays were designed (37, 45) (Figure 4A). The first two assays were used to determine whether rhein affected the adhesion and binding of EB particles to cell membranes, while the third assay was used to determine whether rhein inhibited *C. trachomatis* during its replication stage. The influence-on-post-adsorption assay showed a significant inhibitory effect of rhein (Figures 4B–D). Our previous study demonstrated that the extracellular signaling-regulated kinase (ERK)/ribosomal S6 kinase (RSK) signaling pathway was important in *C. trachomatis* infection (46). To investigate the mechanism of action of rhein in *C. trachomatis* infection, the protein of ERK and RSK were performed by western blotting in the current study on *C. trachomatis*-infected cells treated with rhein for different times post-infection. P-RSK expression was up-regulated at 12 h after *C. trachomatis* infection in the presence or absence of rhein. P-ERK and P-RSK were both down-regulated in the presence of rhein at 36 h and 48 h post-infection (Figures 4E,F). The total ERK and RSK remain constant. Moreover, in cell lines of murine (McCoy) and primate (Vero) origin, the antibacterial activity of rhein was also exerted during the replication stage of *C.trachomatis* (47) (Supplementary Figure S1). These observations suggest that the inhibitory activity of rhein may not be host cell-specific and that rhein may regulate host cells and change the environment to inhibit *C. trachomatis* replication.

### Rhein and AZM had a synergistic inhibitory effect against *C. trachomatis*

**Figure 5:**
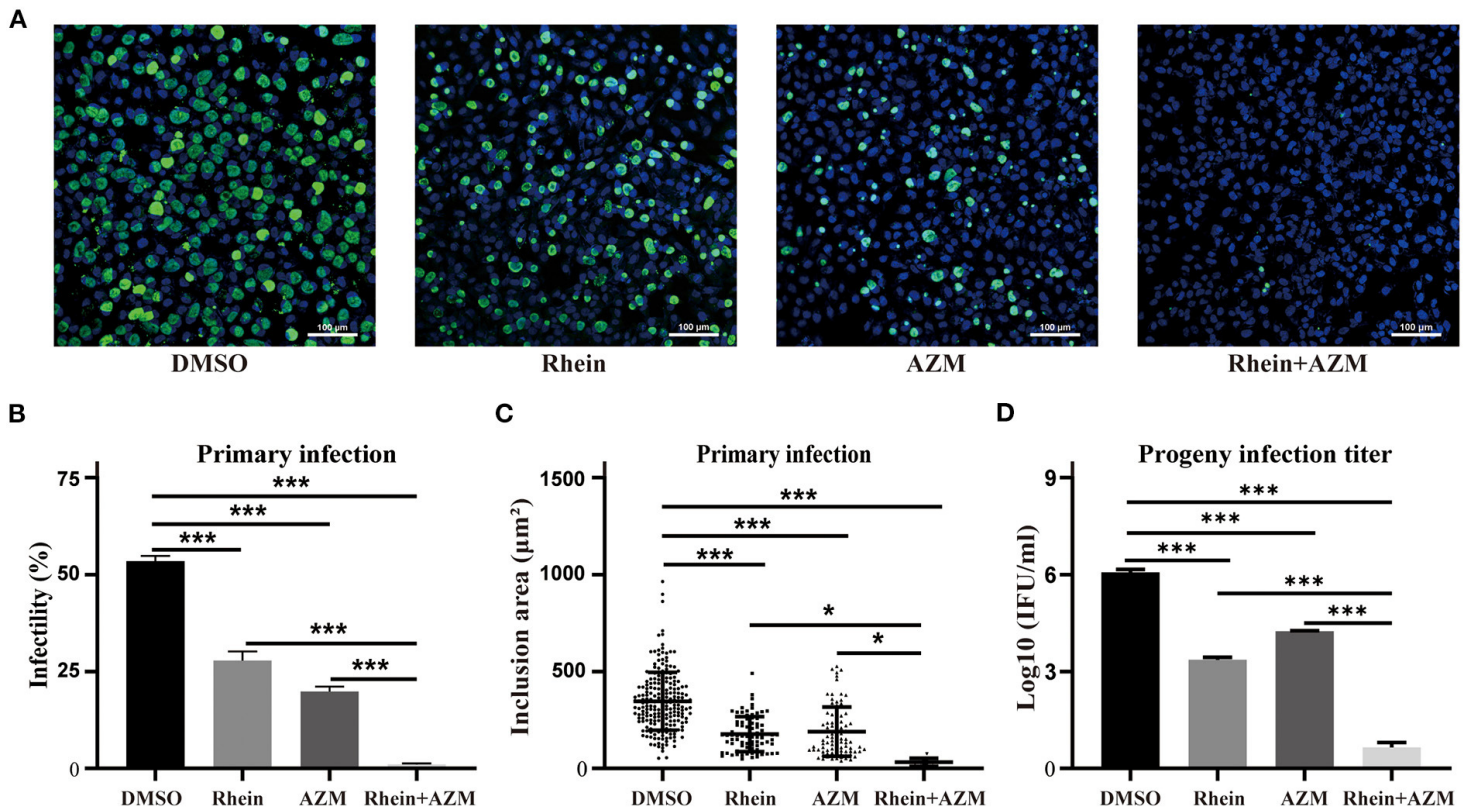
Rhein and AZM combined had synergistic inhibitory effects on *C. trachomatis*. **(A)** Immunofluorescence images (×200 magnification; scale bars, 100 μm) of control (DMSO), 20 μM rhein, 0.005 mg/l AZM, and 20 μM rhein + 0.005 mg/l AZM treatments are shown from left to right. **(B)** Infectivity, **(C)** inclusion area, and **(D)** infectious progeny titer of *C. trachomatis* according to treatments. Data represent the mean ± standard deviation from three independent experiments. *p< 0.05, **p < 0.01, ***p< 0.001.

AZM is a first-line drug for treating *C. trachomatis* infections, but treatment failure has been reported (15, 17). Although rhein alone impaired growth of *C. trachomatis*, an experiment was conducted to investigate whether rhein and AZM had a synergistic suppressive effect on *C. trachomatis* infection. Sub-inhibitory concentrations of 20 μM rhein and 0.005 μg/ml AZM were tested. The infectivity, the area of inclusions and infectious progeny of *C. trachomatis* were reduced by the two individual treatments (rhein alone and AZM alone) (Figure 5). However, a greater inhibitory effect on *C. trachomatis* replication was observed when rhein was combined with AZM compared with rhein alone and AZM alone (Figure 5). Thus, the combination of rhein and AZM potentially has great clinical value.

### Rhein combined with AZM inhibited *C. trachomatis* infection in mouse models

**Figure 6:**
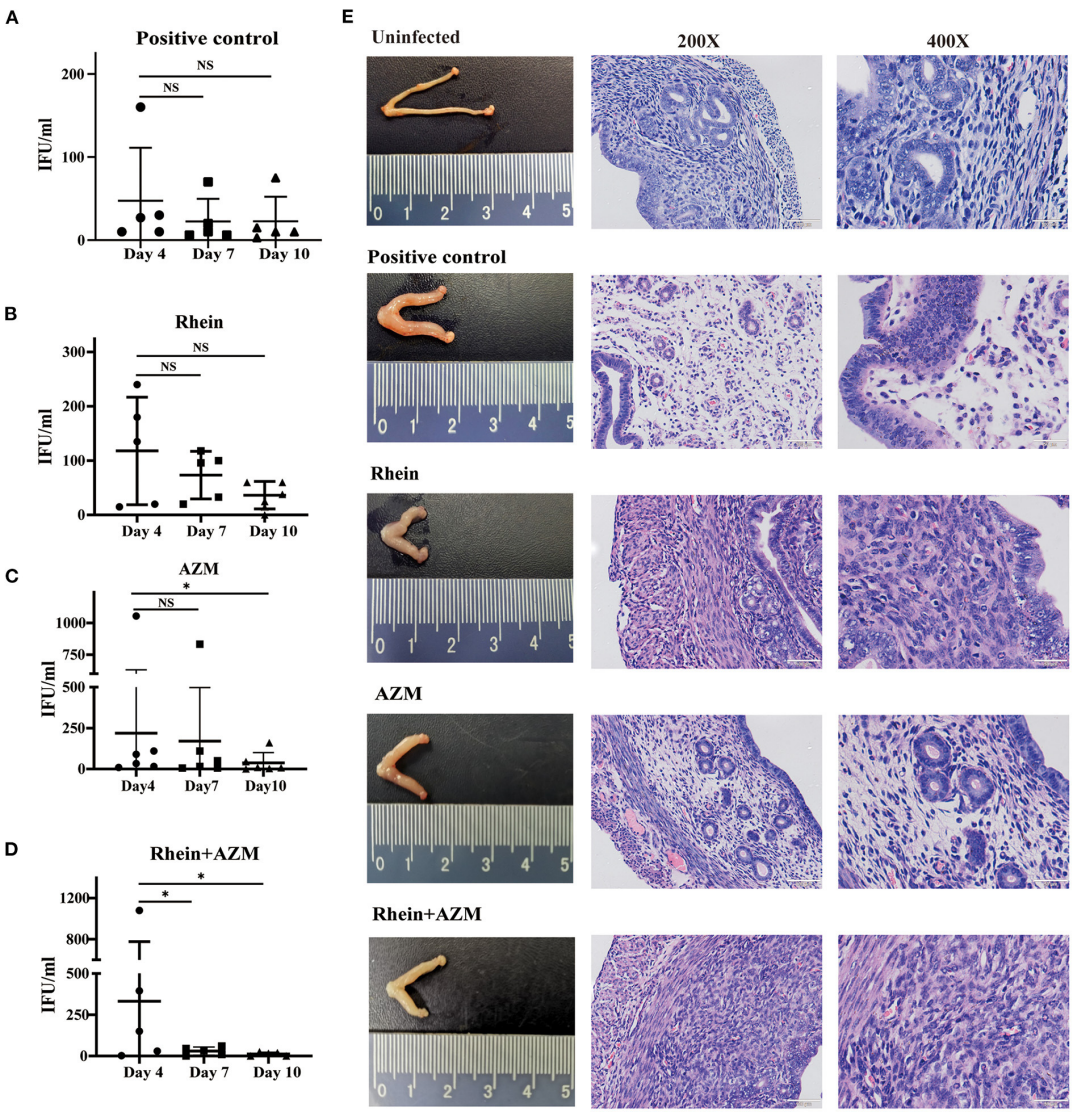
Rhein combined with AZM inhibited *C. trachomatis* infection in mouse models. Vaginal swabs were taken on days 4, 7, and 10 after infection for cell culture and determination of the number of inclusion bodies. **(A)** Positive control group. **(B)** Rhein treatment group. **(C)** AZM treatment group. **(D)** AZM + rhein treatment group. **(E)** Pathological changes in the gross morphology of the uterus (×200 magnification; scale bars, 100 μm. ×400 magnification; scale bars, 50 μm). The nonparametric Wilcoxon test was used for statistical analysis. NS, not significant; *p< 0.05, **p < 0.01, ***p< 0.001.

The *in vitro* experiments demonstrated that rhein effectively inhibited *C. trachomatis* infection, and when combined with AZM, there was a synergistic inhibitory effect. The inhibitory effect of rhein on *C. trachomatis* was therefore tested *in vivo* in a mouse model. Six-week-old female BALB/c mice were infected with *C. trachomatis* serovar D, then DMSO, AZM, rhein and AZM + rhein were administered orally from day 4 to day 10 post-infection. Vaginal swabs were taken on days 4, 7 and 10 for cell culture and to determine the number of infectious progenies. The number of infectious progenies in the DMSO control and rhein-treated group was not significantly different between days 4, 7, and 10 (Figures 6A,B). However, the number of infectious progenies in the AZM-treated group decreased significantly from day 4 to day 10 (Figure 6C), and the number of infectious progenies in the AZM + rhein treatment group decreased significantly from day 4 to day 7 (Figure 6D). Murine tissues were examined by hematoxylin and eosin (H&E) staining on day 22 after *C. trachomatis* infection. Edema and hypertrophy were observed in the uterus of infected mice (Figure 6E), but the uterine edema was relieved in the rhein and/or AZM treatment groups. There were no pathological changes in the heart, liver, spleen, or kidney of mice in any treatment group as revealed by H&E staining (Supplementary Figure S2).

## Discussion

Rhein significantly inhibited *C. trachomatis* replication across various serovars and in HeLa, McCoy and Vero host cells. In combination with AZM, rhein exerted a synergistic suppressive effect on *C. trachomatis* infection, both *in vitro* and *in vivo*. In addition, rhein may regulate host cells and change the environment to inhibit *C. trachomatis* replication. Taken together, the findings of this study suggest that rhein may be a potential treatment for *C. trachomatis* infection.

Rhein was previously reported to have effective antibacterial and antiviral activity against *S. aureus, Helicobacter pylori*, influenza A virus, and hepatitis B virus (HBV) (33, 36, 46). The mechanism of action of rhein was shown to involve direct impairment of pathogens or regulation of host cell signaling pathways. Rhein increased the transcription of genes encoding the iron-regulated surface determinants system and genes involved in the ribonucleotide reductase systems of *S. aureus* (33). In addition, rhein exerted its antimicrobial activity against *S. aureus* by reducing the transcription of genes responsible for anaerobic respiration and fermentation (33). Rhein inhibited DNA polymerase activity in HBV (48). In above studies, the mechanisms of action of rhein involve direct impairment of pathogens. However, rhein also significantly inhibited influenza A virus-induced oxidative stress and decreased influenza A virus-induced expression of Toll-like receptor 2 (TLR2), TLR3 and TLR4. Moreover, rhein suppressed influenza A virus-induced activation of host signaling pathways including the Akt, p38/JNK MAPK and NF-κB pathways in A549 cells (36). In the current study, rhein did not have a direct inactivating effect on *C. trachomatis*, but rather inhibited this pathogen in a post-adsorption replication stage. *C. trachomatis* is an intracellular pathogen that is heavily dependent on host cells, thus the mechanism of rhein inhibition of *C. trachomatis* may be similar to that of influenza A virus whereby host cells are regulated to affect the growth and development of pathogens (49).

Rhein has multiple targets and consequently regulates multiple pathways at the molecular level, including the MAPK signaling pathway, the PI3K-AKT signaling pathway, and the Wnt signaling pathway (31). Among these pathways involved in the pharmacological activity of rhein, the MAPK signaling pathway can be considered one of the most interactive pathways and rhein can regulate the Ras/Raf/MEK/ERK pathway to inhibit the phosphorylation of ERK1/2 (50, 51). The ERK pathway is considered crucial in cell proliferation and migration and RSK is an important downstream effector of the Ras/Raf/MEK/ERK signaling pathway (52, 53). Phosphorylated substrates of RSK are involved in diverse cellular processes including gene transcription, protein synthesis, cell cycle regulation, and cell survival (54, 55). ERK signaling pathways are the most prominent kinase signaling network utilized by *C. trachomatis* and have been characterized as being instrumental in nutrient acquisition, host cell apoptosis resistance, immune responses, and even pathology associated with chlamydial infections (56–58). Moreover, our previous study suggested that ERK/RSK may be a novel target for *C. trachomatis* therapeutics (46). In this study, phosphorylated ERK/RSK was reduced upon exposure to rhein, suggesting that rhein may inhibit *C. trachomatis* infection by regulating the ERK/RSK pathway.

In the process of infectious disease treatment and drug development, host-directed therapy (HDT) is a novel strategy for treating bacterial and viral infections. Biological products or small molecules are used to interfere with replication or persistence of the pathogen by regulating host factors (59). Currently, small-molecule drugs have been proposed for the management of tuberculosis, HBV and HIV by HDT (60–62). *C. trachomatis* development requires host cell energy and nutrients and may therefore be a suitable pathogen for the development of HDT (63–65). The small molecule mycophenolate mofetil was recently demonstrated to effectively inhibit *C. trachomatis* growth by targeting the rate-limiting enzyme inosine-5′-monophosphate dehydrogenase in the biosynthesis of guanine nucleotides in host cells (66). In addition, our research team reported that inhibitors targeting ERK/RSK had potential in the treatment of *C. trachomatis* infection (46). Findings from the current study indicated that rhein may regulate host cells and change the environment to inhibit *C. trachomatis* replication. Moreover, rhein and AZM had a synergistic inhibitory effect on *C. trachomatis in vitro* and *in vivo*. Rhein may therefore be a potential drug for a HDT strategy of managing chlamydial infections.

Although rhein was demonstrated to inhibit *C. trachomatis* infection, the precise molecular mechanism of rhein on *C. trachomatis* has not yet been elucidated. Current research suggests that rhein inhibits *C. trachomatis* survival most likely through targeting host factors. Future work will explore the molecular mechanism by which rhein affects *C. trachomatis* replication.

In summary, this study provided evidence that rhein reduced *C. trachomatis* replication in *vitro* and *in vivo* and indicated that rhein may have potential in drug development for the treatment of *C. trachomatis*.

## Data availability statement

The raw data supporting the conclusions of this article will be made available by the authors, without undue reservation.

## Ethics statement

The animal study was reviewed and approved by the Ethics Committee of South China Agricultural University (SCAU, Guangzhou, China and approval number: 2020c035).

## Author contributions

XY and QX performed most of the experiments in this study and jointly wrote the draft manuscript. WC and ZM were responsible for the initial data analysis. XS and LM compiled figure preparation and statistical analysis. JO and YL provided experimental assistance and constructive comments to this study. HZ and YX had the leading contribution to the design of studies and interpretation of the whole dataset. All authors read and approved the manuscript.

## Funding

This work was supported by grants from the National Natural Science Foundation of China (81974307), the Natural Science Foundation of Guangdong Province (2018A030313662, 2019A1515011827, and 2021A1515012255), Research Foundation of Department of Education of Guangdong Province (2021KTSCX014), Science and Technology Program of Guangzhou (202201011579), and Guangdong Province Bureau of Traditional Chinese Medicine Scientific Research Project (20211276).

## Conflict of interest

The authors declare that the research was conducted in the absence of any commercial or financial relationships that could be construed as a potential conflict of interest.

## Publisher's note

All claims expressed in this article are solely those of the authors and do not necessarily represent those of their affiliated organizations, or those of the publisher, the editors and the reviewers. Any product that may be evaluated in this article, or claim that may be made by its manufacturer, is not guaranteed or endorsed by the publisher.
