# Supplementary material for: Rhein inhibits Chlamydia trachomatis infection by regulating pathogen-host cell
Source: Front Public Health. 2022 Sep 26;10:1002029. doi: 10.3389/fpubh.2022.1002029 (PMC9552556; doi:10.3389/fpubh.2022.1002029)
Supplement: Supplementary file 1 [file Data_Sheet_1.PDF]

Supplementary Figures

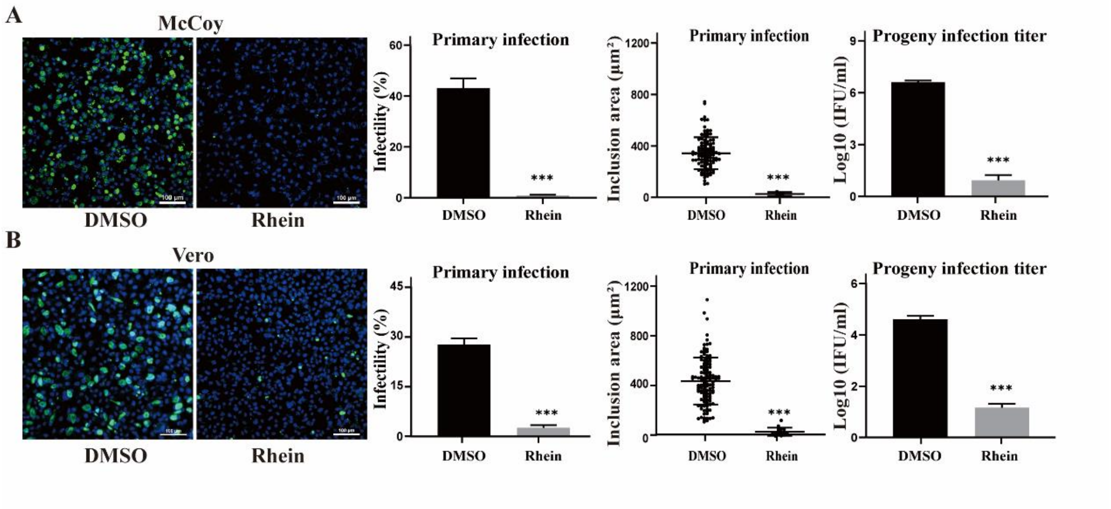

Fig. S1 Rhein repressed *C. trachomatis* infection in McCoy and Vero cells. (A) McCoy and **(B)** Vero cells were infected with *C. trachomatis* serovar D and cultured in medium with 40 μM rhein or DMSO for 48 h. Fluorescent images were captured at ×200 magnification. Scale bars, 100 μm. Data represent the mean ± standard deviation from three independent experiments. Fisher's exact test (Infectivity), a Kruskal–Wallis test followed by Dunn's multiple comparisons test (Inclusion area), and an unpaired *t*-test (EB titer) were used for statistical analyses. *$p < 0.05$, **$p < 0.01$, ***$p < 0.001$.

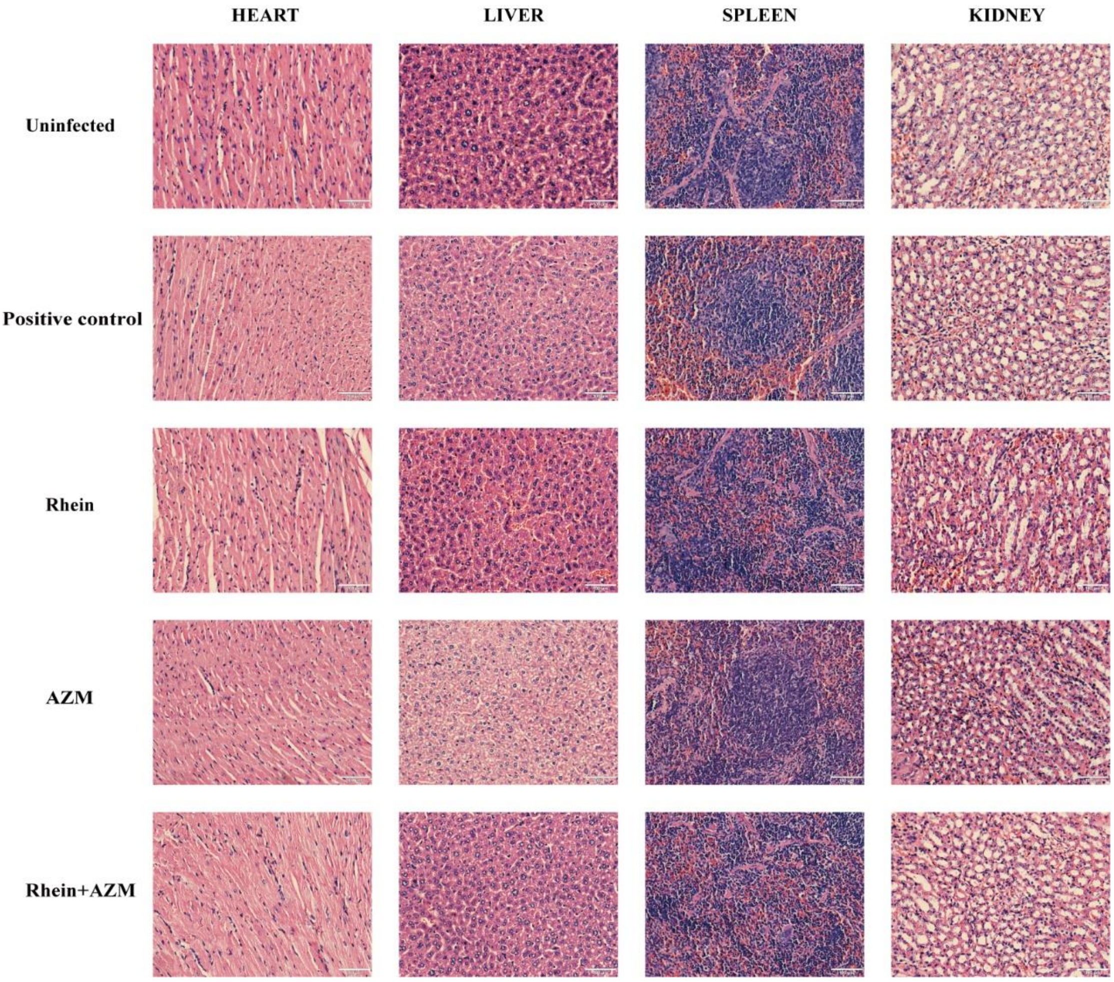

Fig. S2 *In vivo* toxicity of rhein. Hearts, livers, spleens, and kidneys of mice were harvested and stained with hematoxylin and eosin. Treatments top to bottom: uninfected, positive control (DMSO), rhein, azithromycin (AZM), and rhein + AZM. Scale bars, 100 μm.
